# Protective and Risk Factors for Mental Distress and Its Impact on Health-Protective Behaviors during the SARS-CoV-2 Pandemic between March 2020 and March 2021 in Germany

**DOI:** 10.3390/ijerph18179167

**Published:** 2021-08-31

**Authors:** Donya Gilan, Markus Müssig, Omar Hahad, Angela M. Kunzler, Simon Samstag, Nikolaus Röthke, Johannes Thrul, Frauke Kreuter, Michael Bosnjak, Philipp Sprengholz, Cornelia Betsch, Daniel Wollschläger, Oliver Tüscher, Klaus Lieb

**Affiliations:** 1 Leibniz Institute for Resilience Research (LIR), 55131 Mainz, Germany; markus.muessig@lir-mainz.de (M.M.); omar.hahad@unimedizin-mainz.de (O.H.); angela.kunzler@lir-mainz.de (A.M.K.); s.samstag@gmx.net (S.S.); oliver.tuescher@unimedizin-mainz.de (O.T.); klaus.lieb@lir-mainz.de (K.L.); 2 Department of Psychiatry and Psychotherapy, University Medical Center of the Johannes Gutenberg University Mainz, 55131 Mainz, Germany; nikolaus.roethke@unimedizin-mainz.de; 3 Department of Personality Psychology and Psychological Assessment, Johannes Gutenberg University Mainz, 55122 Mainz, Germany; 4 Department of Cardiology, Cardiology I, University Medical Center of the Johannes Gutenberg University Mainz, 55131 Mainz, Germany; 5 Johns Hopkins Bloomberg School of Public Health, Baltimore, MD 21205, USA; jthrul@jhu.edu; 6 Ludwig Maximilians University of Munich, 80539 Munich, Germany; fkreuter@umd.edu; 7 University of Maryland, College Park, MD 20742, USA; 8 Leibniz Institute for Psychology (ZPID), 54296 Trier, Germany; mb@leibniz-psychology.org; 9 Center for Empirical Research in Economics and Behavioural Sciences, University of Erfurt, 99089 Erfurt, Germany; philipp.sprengholz@uni-erfurt.de (P.S.); cornelia.betsch@uni-erfurt.de (C.B.); 10 Institute of Medical Biostatistics, Epidemiology and Informatics (IMBEI), University Medical Center of the Johannes Gutenberg University Mainz, 55131 Mainz, Germany; wollschlaeger@uni-mainz.de

**Keywords:** resilience, protective factors, pandemic, SARS-CoV-2, mental distress, protective behavior

## Abstract

The severe acute respiratory syndrome coronavirus 2 (SARS-CoV-2) pandemic is posing a global public health burden. These consequences have been shown to increase the risk of mental distress, but the underlying protective and risk factors for mental distress and trends over different waves of the pandemic are largely unknown. Furthermore, it is largely unknown how mental distress is associated with individual protective behavior. Three quota samples, weighted to represent the population forming the German COVID-19 Snapshot Monitoring study (24 March and 26 May 2020, and 9 March 2021 with >900 subjects each), were used to describe the course of mental distress and resilience, to identify risk and protective factors during the pandemic, and to investigate their associations with individual protective behaviors. Mental distress increased slightly during the pandemic. Usage of cognitive reappraisal strategies, maintenance of a daily structure, and usage of alternative social interactions decreased. Self-reported resilience, cognitive reappraisal strategies, and maintaining a daily structure were the most important protective factors in all three samples. Adherence to individual protective behaviors (e.g., physical distancing) was negatively associated with mental distress and positively associated with frequency of information intake, maintenance of a daily structure, and cognitive reappraisal. Maintaining a daily structure, training of cognitive reappraisal strategies, and information provision may be targets to prevent mental distress while assuring a high degree of individual protective behaviors during the COVID-19 pandemic. Effects of the respective interventions have to be confirmed in further studies.

## 1. Introduction

The virus SARS-CoV-2, causing COVID-19, a disease first described in China in late 2019, has led to a worldwide pandemic with currently over 173 million confirmed cases and over 3.7 million deaths (8 June 2021; [1]). To control the spread of the disease, many countries have put restrictions in place that heavily impacted their economies as well as everyday life. These restrictions combined with the pandemic itself have led to increased mental distress caused by, among other things, fear of job loss, fear of losing loved ones, financial problems, social isolation, closed educational institutions, and restricted access to healthcare services [2,3,4,5,6]. A key question in the current pandemic that remains under-researched from a resilience perspective is: which individual characteristics contribute to this mental distress and which may prevent it, that is, may foster resilience as the maintenance or fast recovery of mental health during or after stressful life events [7]?

Compared with the global situation with steadily rising SARS-CoV-2 cases and more cases per capita in many countries, the impact of the SARS-CoV-2 pandemic has been less pronounced in Germany than, for example, in Italy, Spain, Brazil, or the United States [1]. Nevertheless, in our previous study that assessed mental distress in three representative cross-sectional samples of the German population between 24 March and 21 April 2020, during the first wave of the pandemic, we found increased levels of mental distress (i.e., symptoms of depression, anxiety, loneliness, and hopelessness) compared with pre-pandemic data [8]. This is in line with a large number of systematic reviews and meta-analyses that have been published on the mental distress caused by the SARS-CoV-2 pandemic so far (e.g., [4,5,6,9,10]). For example, Kunzler and colleagues [9] compared mental distress during the SARS-CoV-2 pandemic in the general population (50 international studies) with pre-pandemic data and found evidence for an increase in symptoms of anxiety (d = 0.40) and depression (d = 0.67), but not in other mental health outcomes (e.g., stress). Similarly, Prati and Mancini [10] focused on the psychological impact of lockdown on the general population and reported small effects on depression (g = 0.15) and anxiety (g = 0.17), while there was no evidence for an impact on other mental health outcomes. Although the evidence base on the mental health impact of the COVID-19 pandemic has improved in the past few months, with an increasing number of longitudinal studies (e.g., comparing pre-pandemic data on mental health outcomes with data collected during the pandemic; [11,12]), the majority of existing studies suffer from methodological weaknesses such as non-representative samples, cross-sectional designs with a single data collection, and the use of non-validated questionnaires. Additionally, most previous studies focused on acute effects on mental distress, while data on the long-term consequences of the pandemic with its different waves and associated consequences are lacking.

Factors contributing to or mitigating mental distress from SARS-CoV-2 have also been researched in some existing studies. Reported risk factors for mental distress in response to SARS-CoV-2 include female gender [9,11,12], working as a healthcare professional [8,13], previous diagnoses of mental illness [9,14], symptoms of a pre-existing physical condition [15], younger age [11,12], relatives who are infected with COVID-19 [15,16], concerns about getting infected [9], being under lockdown or quarantine [17], and loneliness [15,17,18]. Protective factors for mental distress, on the other side, have been studied less comprehensively, and some of these protective factors include higher age [9], socioeconomic stability [9,12], education [9], spiritual well-being [15], sociability [19], and social support [17].

Apart from these mostly sociodemographic factors, only a few studies have evaluated the use of psychological coping strategies during the pandemic. For example, Veer et al. [20] identified a positive appraisal style as a protective coping style, while Shamblaw, Rumas, and Best [21] found that avoidance-oriented coping styles were associated with higher depression and anxiety ratings. Nevertheless, little is known about protective coping behaviors in light of the pandemic.

Moreover, there is limited evidence on associations between psychological factors and individual health-protective behaviors during the pandemic such as wearing masks in public spaces, hand-hygiene, and keeping a physical distance of 1.50 m to other people, which are considered crucial to reducing the spread of SARS-CoV-2. Harper et al. [22] reported that fear of SARS-CoV-2 was associated with individual health behavior, while Allington et al. [23] reported that beliefs in conspiracy theories and the use of social media as a source of information were factors associated with non-adherence to recommendations for protective behaviors. Even though preliminary evidence suggests the importance of psychological factors, such as fear and information behavior, for the adherence to individual protective behaviors, the evidence is far from conclusive.

The aim of this study was to extend our knowledge about risk and protective factors of mental distress connected to the SARS-CoV-2 pandemic as well as psychological factors contributing to the adherence to recommended individual protective behaviors.

**Figure 1:**
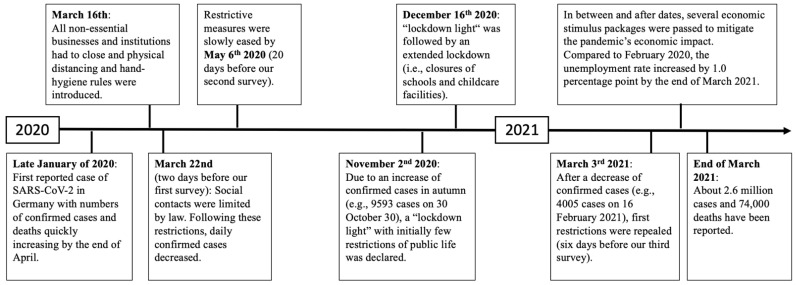
Progress of the pandemic and restrictions in Germany between March 2020 and March 2021.

Specifically, we wanted to know (1) how mental distress and risk and protective factors changed from the first wave in 2020 to the third wave in 2021, (2) which risk and protective factors were most strongly associated with mental distress, and (3) how mental distress and psychological factors were associated with adherence to individual health-protective behaviors. Since the measures imposed by the German government are likely important to the adherence to individual health-protective behaviors and mental distress, we show the timeline of the most important measures and dates to our research in Figure 1. To answer these questions, we used data from three large cross-sectional samples (*N* > 900 each), representative of the German population on key variables, collected between March 2020 and March 2021 at different stages of the pandemic.

## 2. Methods

### 2.1. Participants

This investigation is part of the “COVID-19 Snapshot Monitoring” (COSMO) study, which was initiated in Germany on 3 March 2020, to gain behavioral and cognitive insights into the German population’s reaction to the SARS-CoV-2 pandemic [24]. Mental distress and related protective and risk factors of mental distress during the SARS-CoV-2 outbreak were measured several times on a quota-panel representative of the German population regarding age and gender (24 March 2020; 26 May 2020; 9 March 2021). The quota samples were drawn from the panel supplier *respondi*’s actively managed panel, recruited via online and offline campaigns as well as cooperation partners to achieve a representation of the German population on key variables. The questionnaire was sent to around 5000 people at each starting date, to be completed within 48 h. Samples are therefore distinct, non-overlapping, and cross-sectional, that is, individuals were not repeatedly assessed. The sample from 24 March 2020 has already been described in Gilan et al. [8].

### 2.2. Study Variables

#### 2.2.1. Mental Distress

Here, we understand mental distress as the degree to which respondents report experiencing symptoms of different mental illnesses, such as depressive or anxious symptoms. Mental distress was measured with five items taken from three different questionnaires to be rated on a four-point Likert scale (1 = never or less than once a week, 4 = five to seven days a week), to allow for an approximation of a broad range of factors contributing to mental distress (see also [8]). Items were compiled from the German version of the Generalized Anxiety Questionnaire-7 ([25]; single item: “I felt nervous, anxious, or tense”), the German version of the Center for Epidemiologic Studies-Depression (CES-D) scale ([26]; three items: “I felt depressed”, “I felt lonely”, “I felt hopeful about the future”), and the German version of the Impact of Event Scale-Revised ([27]; one reworded item: “Reminders of my experiences with the corona pandemic caused me to have physical reactions, such as sweating, trouble breathing, nausea, or a pounding heart”). Mental distress was then calculated as the mean of the five items. Internal consistency was between α = 0.77 and α = 0.79. For a full list of the items used, see Supplementary Table S1. Data on mental distress from the first assessment date have been published previously and served, combined with two additional assessment dates, as the basis for this investigation into protective and risk factors as well as consequences for individual health-protective behaviors (see [8]).

#### 2.2.2. Protective and Risk Factors

Self-reported resilience was measured by the German version of the Brief Resilience Scale, which measures the ability to recover from stress as a proxy measure of resilience on a 5-point Likert scale (BRS; [28,29]). Internal consistency was between α = 0.81 and α = 0.85. Since no questionnaires were available to assess pandemic-specific behavior as well as behavioral and cognitive coping strategies during a pandemic, new items were created by members of the COSMO group. We measured SARS-CoV-2-specific reappraisal, which refers to the positive reappraisal of aspects of the pandemic such as future prospects, social interaction, which refers to the degree to which people communicate with their family or support neighbors, and maintaining a daily structure, which refers to the degree to which people stick to a daily routine. All items were answered on a 7-point Likert scale. For this purpose, four items assessed SARS-CoV-2-specific reappraisal (e.g., “During the pandemic, I learned important and useful lessons for my life.”) with an internal consistency between α = 0.77 and α = 0.79. Three items assessed social interactions (e.g., “I use phone and digital services to communicate with my family and friends.”) with an internal consistency between α = 0.40 and α = 0.51. Two items assessed maintaining a daily structure (e.g., “I have a plan for everyday life regarding sleep, work, and physical activities.”) with an internal consistency between α = 0.30 and α = 0.37. One item assessed the frequency of information intake (“How many times per day do you seek information regarding the coronavirus/SARS-CoV-2?”). Scale values were calculated as the mean value of all corresponding items. For details, see Supplementary Table S1.

#### 2.2.3. Individual Protective Behaviors

Individual protective behaviors, namely wearing masks in public spaces, hand hygiene, and keeping a physical distance of 1.5 m, were included in the survey as items with a five-point Likert scale on 26 May 2020 and 9 March 2021. The scale points were dichotomized into the categories non-adherence for answers “never”, “rarely”, or “sometimes” and adherence for answers “often” or “always”.

### 2.3. Analyses

To investigate changes in mental distress as well as risk and protective factors over time, we conducted one-way analyses of variance (estimated effect sizes η^2^: small > 0.01, medium > 0.06, large > 0.14). To identify the protective and risk factors most strongly associated with mental distress, we first conducted correlation analyses (estimated effect sizes *r*: small > 0.10, medium > 0.30, large > 50; [30]), followed by multiple regression analyses with overall resilience, SARS-CoV-2-specific reappraisal, maintaining a daily structure, social interaction, and frequency of information intake as predictors, controlling for age since it was the only sociodemographic variable that was significantly different between the samples (estimated effect sizes *R*^2^: small > 0.02, medium > 0.13, large > 0.26; [30]). We conducted both the correlation analyses as well as the regression analyses separately for each date to identify predictors that were consistently linked to mental distress at all survey dates.

As the legal situation changed in between dates, we only analyzed data from the last survey date (9 March 2021) to investigate factors associated with individual protective behaviors. Gender differences in adherence to individual protective behaviors were assessed via a χ^2^ test. Associations between individual protective behavior as a binary variable (adherence vs. non-adherence) and the other study variables were calculated as point-biserial correlations. Due to the high number of analyses performed and their largely exploratory nature, a significance level of α = 0.005 was applied in accordance with Benjamin et al. [31]. Statistical analyses were performed in IBM SPSS Statistics version 27.0 (IBM Corporation, Armonk, NY, USA) [32]. Original data can be obtained from the corresponding author upon request.

## 3. Results

Sample characteristics regarding age, gender, education, number of people in a household, immigrant status, and healthcare personnel are summarized in Table 1. On the first date (March 2020), 1114 subjects (mean age = 50.45, SD age = 18.42, 51.35% female) participated, on the second date (May 2020), 925 subjects (mean age = 44.83, SD age = 15.49, 47.68% female) participated, and on the third date (May 2021) 994 subjects participated (mean age = 45.03, SD age = 15.37, 49.70% female). The assessment of sample comparability between the three samples showed significant, but small, differences in sample age and number of household members of respondents.

### 3.1. Mental Distress and Protective and Risk Factors during the Pandemic

As already reported in our previous publication, the sample at the first assessment (24 March 2020) showed a small increase in mental distress compared with before the pandemic [8]. One-way ANOVA revealed that mental distress increased significantly but with a very small effect size of η^2^ = 0.006 (see Table 2 and Supplementary Figure S1). Post-hoc Tukey tests revealed that this increase occurred between the second (26 May 2020) and the third assessment date (9 March 2021).

While self-reported resilience remained stable across all three survey dates, the usage of SARS-CoV-2-specific reappraisal strategies, the ability to maintain a daily structure, and the amount of social interaction decreased during the pandemic with small effect sizes. The frequency of information intake regarding SARS-CoV-2 decreased between March 2020 and May 2020 and increased between May 2020 and March 2021. In sum, mental distress, starting from a slightly increased level compared with before the pandemic, further increased during the pandemic, while the use of cognitive and behavioral strategies as potential protective factors decreased over time.

### 3.2. Associations between Psychological Factors and Mental Distress

Further analyses revealed several cognitive and behavioral strategies associated with mental distress during the SARS-CoV-2-pandemic (Table 3). At all assessment dates, resilience, the usage of SARS-CoV-2-specific reappraisal, and maintaining a daily structure were associated with reduced mental distress. Social interactions showed a negative correlation with mental distress at the third assessment date only, and frequency of information intake regarding SARS-CoV-2 per day was not correlated to mental distress at any assessment date.

Since we found several strategies linked to mental distress, we proceeded to examine their relative importance via regression analyses, controlling for age (Table 4). In all three samples, the included predictors explained over 30% of the sample variance (all *p* < 0.005) in mental distress. Consistently, resilience (range of β-weights: −0.431 to −0.478), maintaining a daily structure (range of β-weights: −0.159 to −0.191), and SARS-CoV-2-specific reappraisal (range of β-weights: −0.074 to −0.191) were significantly associated with reduced mental distress. Social interaction and the frequency of information intake were not associated with mental distress.

### 3.3. Mental Distress and Other Predictors of Individual Health-Protective Behavior

Compliance with individual health-protective behaviors at the third assessment date (March 2021) was overall high as 85.9% of the sample reported keeping a physical distance of 1.50 m, 92.0% of the sample reported wearing masks in public spaces, and 75.1% of the sample reported following hand hygiene recommendations. Overall, women were significantly more likely than men to adhere to physical distancing and hand hygiene recommendations (distancing: χ^2^(1) = 15.26, *p* ≤ 0.001, *V* = 0.124; hand hygiene: χ^2^(1) = 16.55, *p* ≤0.001, *V* = 0.129), but not more likely to adhere to mask rules (Mask: χ^2^(1) = 7.58, *p* = 0.006, *V* = 0.087).

Point-biserial correlations revealed that increased mental distress was associated with less distancing and mask-wearing behavior, with a trend for a negative correlation between mental distress and hand hygiene behavior as well. SARS-CoV-2-specific reappraisal, maintaining a daily structure, social interaction, and the frequency of information intake regarding SARS-CoV-2 were positively associated with almost all individual protective behaviors. We found no evidence for a correlation between resilience and any of the three protective behaviors (Table 5).

## 4. Discussion

The present study found that all three examined samples of the German population showed increased mental distress as compared with the time before the SARS-CoV-2 pandemic. This is in line with previous cross-sectional studies summarized in several systematic reviews [4,5,6,8,9]. Furthermore, our study showed that this increased mental distress remained stable or further increased over time and the different stages of the pandemic, with our study being the first to demonstrate further increased mental distress during the third wave of the pandemic in spring 2021.

The increased mental distress found in our study was negatively correlated with strategies that potentially protect against mental distress, such as maintaining a daily structure or a SARS-CoV-2-specific cognitive reappraisal style. However, due to the repeated cross-sectional nature of our data, we cannot draw conclusions about any causal relationships. A potential explanation might be that the use of these behavioral and cognitive coping strategies was made more difficult by the declared constraints of daily life or other developments of the pandemic. For example, maintaining a daily structure is severely hampered by lockdown restraints. Additionally, cognitive reappraisal may be more difficult when, for example, incidence numbers sharply increase, or the media are reporting frightening new findings about the pandemic. On the other hand, one could also argue that the increased mental distress itself may be the cause for not using these strategies. For example, maintaining a daily structure may require a certain level of positive mood, energy, and drive and may be limited by increasing exhaustion. High mental distress may interfere with maintaining structured daily activities. Furthermore, mental distress may also be affected by other factors like existential and financial problems (note that the unemployment rate increased in Germany during the last year [33,34]), which may become increasingly severe over time and can potentially be only poorly managed by the protective strategies described. In particular, worries about one’s economic situation, such as short-term work, unemployment, or bankruptcy, may contribute to mental distress [35]. These factors may lead to an enduring degree of dejection, hopelessness, and loneliness among affected individuals. Further studies should also take these economic aspects into account and more closely investigate their effects on mental distress.

Regarding protective coping strategies against mental distress, maintaining a daily structure, SARS-CoV-2-specific cognitive reappraisal, and the self-reported level of resilience were the most important factors in all three samples. The last point in particular confirms our previous research, which already found evidence that resilient individuals reported less mental distress over the course of the pandemic [36]. It is also important to note that resilience explained additional variance in mental distress beyond maintaining a daily structure and cognitive reappraisal. This may be an indicator that resilience includes further coping strategies and protective factors in addition to maintaining a daily structure and cognitive reappraisal. Further studies may want to expand this important area of research by investigating more precisely how resilient people cope.

On the other hand, our analyses could not confirm the frequency of information intake and social interactions as protective factors. This is especially surprising for social interactions because it is a frequently reported protective factor for mental health (e.g., [17]). An explanation might be our operationalization of social support, as we focused on concrete social support behaviors (i.e., offering support to neighbors and relatives) rather than general social support or the number of social interactions without specific descriptions. Future studies should also include cognitive aspects of social support, such as the perception of received support, which remains a promising protective factor, as well as measures on the frequency of social interactions or more information on the meaning of interaction with family and friends. Another potential aspect could be that the relevance of social interactions fluctuated depending on constraints such as restrictions of contacts because we could find that under these conditions, the correlation between mental distress and social interaction increased in the first and last measurement after the implementation of contact restrictions. Our results regarding the frequency of information intake can be connected to previous research, which highlights this factor as an ambivalent or even dysfunctional coping strategy. For example, Petzold et al. [37] found that participants who spent two hours or more per day thinking about SARS-CoV-2 showed more symptoms of depression and anxiety. The result that the frequency of information intake was enhanced while incidence rates were higher and constraints were declared in March 2020 and March 2021 could be explained by a heightened salience of the topic and the consequences of the constraints (e.g., being home and increased media consumption). Another explanation could be an increased fear of the virus under these conditions. SARS-CoV-2-related fear was not directly assessed in this study but was previously shown to correlate with protective behavior [22]; information intake could also be interpreted similarly when it leads to increased knowledge about the virus.

Another goal of our study was to identify factors contributing to the adherence to individual health-protective behaviors. First, we observed a generally high adherence to protective behaviors such as wearing masks in public spaces, hand hygiene, and keeping a physical distance of 1.5 m. In addition, we identified female gender and coping strategies, such as maintaining a daily structure and especially SARS-CoV-2-specific reappraisal, as protective factors for increased adherence to health-protective behaviors. Mental distress, however, was negatively associated with adherence to protective behaviors.

The relationship found between mental distress and protective behavior is remarkable. We found a generally high adherence to health-protective behavior (e.g., 92% of participants reported wearing masks), which limits the variance in these variables, meaning that significant correlations were difficult to detect. The negative correlation suggesting less health-protective behavior in the case of increased mental distress contrasts with previous studies that, demonstrated predominantly positive correlations between fear (of the pandemic) as a specific form of mental distress and protective behavior (e.g., [22]) and, on a theoretical level, one might also expect that increased mental distress due to the pandemic situation should motivate more protective behavior as an active coping mechanism for this stressful situation. Our results do not confirm this previous finding, which raises the question of how exactly this negative correlation between mental distress and protective behaviors is mediated. The contradicting finding in the current study might be explained by our operationalization of mental distress, which integrated depressive symptoms in addition to symptoms of anxiety. Another possibility could be the restrictions that were in place at the third assessment date (see Figure 1). Heavy restrictions might be linked to increased mental distress and, at the same time, might have reduced the frequency at which people left their homes, decreasing the need to adhere to restrictions. Furthermore, similar to the use of mental coping strategies, it may be the case that a certain level of drive, energy, and positive expectations for the future are necessary for the use of protective behaviors. When the level of depressive symptoms (e.g., hopelessness) gets too high, adhering to protective behaviors may seem overwhelming to individuals.

Another highly important factor for the adherence to health-protective behaviors seemed to be the frequency of information intake. This can be reconciled with further findings of our study group [38], according to which—among other factors—knowledge of the virus (e.g., mechanisms of spread) was associated with the willingness to wear a mask. It could be assumed that this increased knowledge also mediates the present association between the frequency of information intake and the willingness to engage in protective behaviors. If this is the case, public health education programs could be an opportunity to improve adherence to health-protective behaviors. If confirmed by additional research, the results of our study provide a promising starting point for public health education programs and interventions.

## 5. Strengths and Limitations

The strengths of the current study were the unique focus on repeated cross-sectional assessments over one year of the pandemic in representative samples of the German population, protective and risk factors for mental distress, and associations between mental distress and health-protective behaviors. The current study, however, also has several limitations. First, the infection control measures, such as lockdowns and contact restrictions, were not identical in all regions of Germany at the time of the study, which we cannot control for due to the anonymous responses of participants and which may have impacted the study results. Second, since panel members only had two days to answer the survey, it is likely that highly distressed individuals, in particular, did not respond, and our data, therefore, are a lower-bound estimation of mental distress in the German population. Third, the small number of items on the questionnaires is a further limitation since symptoms of depression and anxiety were not assessed in full detail, which would allow for more nuanced conclusions about different dimensions of symptoms. We note, however, that this limitation might also mean that the effect sizes we found are also lower-bound estimates.

## 6. Conclusions

In sum, our results suggest that the COVID-19 pandemic may impact the general population in the form of increased mental distress (i.e., symptoms of depression and anxiety), which was sustained or even increased between March 2020 and March 2021. We cautiously conclude that the maintenance of everyday structures, the training of cognitive coping strategies, and other interventions to foster resilience may be important components to inform the development of appropriate intervention strategies to tackle mental distress during the ongoing pandemic. Interventions addressing these factors should be evaluated in clinical trials to develop the most effective strategies that can contribute to building individual wellbeing and health-protective behavior against pandemics. Additional studies are needed to examine mediating factors behind the increase in mental distress over time. Since mental distress and adherence to health-protective behavior were negatively correlated in our data, interventions to improve mental health may also be effective in reducing the spread of the virus. We also suggest that pandemic preparedness plans broaden their scope to include markers of mental distress in the population to further improve our understanding of mental distress during a pandemic and to enable timely interventions. To foster adherence to health-protective behavior, the evaluation of public health education strategies to improve the information and knowledge that individuals have as key factors for adherence should also be considered as an additional strategy.

## Figures and Tables

**Table 1:** Age and sociodemographic variables of all three samples.

		24 March 2020		26 May 2020		9 March 2021			
*N*		1114		925		994			
		*M*	*SD*	*M*	*SD*	*M*	*SD*	*p*	
Age		50.45	18.42	44.83	15.49	45.03	15.37	<0.001	
		abs	%	abs	%	abs	%	*p*	*V*
Gender	female	572	51.35	441	47.68	494	49.70	0.256	0.03
Education	<10 years	123	11.04	94	10.16	108	10.87	0.840	0.02
	>10 years (without A-levels)	379	34.02	301	32.54	323	32.49		
	<10 years (with A-levels)	612	54.94	530	57.30	563	56.64		
Immigration status	yes	173	15.53	129	13.95	174	17.51	0.245	0.03
Number of people in household	1	331	29.71	233	25.19	236	23.74	0.001	0.07
	2	460	41.29	357	38.59	380	38.23		
	3–4	276	24.78	288	31.14	318	31.99		
	>4	47	4.22	45	4.86	59	5.94		
Healthcare personnel	yes	78	7.00	81	8.76	91	9.15	0.158	0.04

Note. abs = absolute. V = Cramer’s V. Differences in age were calculated via Kruskal–Wallis ANOVA. For all other comparisons, we conducted χ^2^-tests. Note that data for 24 March 2020 have already been published in Gilan et al. [8].

**Table 2:** Changes in mental distress and cognitive and behavioral strategies over time.

	24 March 2020		26 May 2020		9 March 2021				
*N*	1114		925		994				
	M	SD	M	SD	M	SD	F	p	η^2^
Mental distress	1.79	0.60	1.78	0.58	1.88	0.60	9.37	<0.001	0.006
Resilience	3.47	0.60	3.42	0.84	3.35	0.81	4.75	0.005	0.004
SARS-CoV-2-specific reappraisal	5.18	1.16	5.09	1.19	4.88	1.27	17.21	<0.001	0.011
Maintaining a daily structure	5.11	1.44	4.95	1.44	4.60	1.50	32.12	<0.001	0.021
Social interaction	4.42	1.28	4.16	1.34	4.07	1.36	20.64	<0.001	0.013
Frequency of information intake	5.44	1.43	4.81	1.54	5.18	1.49	46.58	<0.001	0.030

Note. Mental distress was measured on a four-point Likert scale. Resilience was measured on a five-point Likert scale. All other variables were measured on a seven-point Likert scale. Due to the heterogeneity of variances between samples, differences in SARS-CoV-2-specific reappraisal were calculated via the Welch F-test. We applied an α level of 0.005. Effect sizes η^2^: small > 0.01, medium > 0.06, large > 0.14.

**Table 3:** Correlations between resilience as well as cognitive and behavioral strategies and mental distress.

	Mental Distress					
	24 March 2020		26 May 2020		9 March 2021	
	*r*	*p*	*r*	*p*	*r*	*p*
Resilience	−0.52	<0.001	−0.56	<0.001	−0.51	<0.001
SARS-CoV-2-specific reappraisal	−0.23	<0.001	−0.26	<0.001	−0.34	<0.001
Maintaining a daily structure	−0.34	<0.001	−0.35	<0.001	−0.31	<0.001
Social interaction	−0.08	0.006	−0.05	0.126	−0.12	<0.001
Frequency of information intake	−0.02	0.483	−0.03	0.430	−0.05	0.121

Note. *r* = Pearson’s correlation coefficient. We applied an α level of 0.005. Effect sizes *r* > 0.10 are small, *r* > 0.30 are medium, and *r* > 0.50 are large.

**Table 4:** Prediction of mental distress by cognitive and behavioral strategies (regression analysis).

	Mental Distress					
	24 March 2020		26 May 2020		9 March 2021	
*R* ^2^	0.338 *		0.372 *		0.334 *	
	β	*p*	β	*p*	β	*p*
Resilience	−0.436	<0.001	−0.478	<0.001	−0.431	<0.001
SARS-CoV-2-specific reappraisal	−0.074	0.008	−0.117	<0.001	−0.191	<0.001
Maintaining a daily structure	−0.191	<0.001	−0.191	<0.001	−0.159	<0.001
Social interaction	−0.006	0.811	0.074	0.012	0.038	0.186
Frequency of information intake	0.086	0.001	0.054	0.055	0.035	0.206

Note. All analyses were controlled for age. We applied an α level of 0.005. Effect sizes *R*^2^ > 0.26 are large. * *p* < 0.001.

**Table 5:** Correlations between mental distress and resilience as well as cognitive and behavioral strategies on the one hand and adherence to individual protective behaviors on the other.

	Physical Distancing		Wearing Masks in Public Spaces		Hand Hygiene	
	*r_pb_*	*p*	*r_pb_*	*p*	*r_pb_*	*p*
Mental distress	−0.11	<0.001	−0.10	<0.005	−0.08	0.011
Resilience	−0.01	0.987	−0.01	0.816	0.02	0.562
SARS-CoV-2-specific reappraisal	0.17	<0.001	0.17	<0.001	0.16	<0.001
Maintaining a daily structure	0.15	<0.001	0.09	<0.005	0.15	<0.001
Social interaction	0.12	<0.001	0.06	0.055	0.15	<0.001
Frequency of information intake	0.24	<0.001	0.15	<0.001	0.19	<0.001

Note. *r*_pb_ = point-biserial correlation. We applied an α level of 0.005. Effect sizes *r* > 0.10 are small, *r* > 30 are medium, and *r* > 0.50 are large.

## Data Availability

The data presented in this study are available on request from the corresponding author.

## Supplementary Materials

The following are available online at https://www.mdpi.com/article/10.3390/ijerph18179167/s1, Table S1 Items used to measure study variables; Figure S1 Changes in mental distress and cognitive and behavioral strategies over time.
