# Supplementary material for: Protective and Risk Factors for Mental Distress and Its Impact on Health-Protective Behaviors during the SARS-CoV-2 Pandemic between March 2020 and March 2021 in Germany
Source: Int J Environ Res Public Health. 2021 Aug 31;18(17):9167. doi: 10.3390/ijerph18179167 (PMC8431087; doi:10.3390/ijerph18179167)
Supplement: Supplementary file 1 [file ijerph-18-09167-s001.zip › ijerph-1316756-supplementary.pdf]

Protective and Risk Factors for Mental Distress and Its Impact on Health-Protective Behaviors during the SARS-CoV-2 Pandemic between March 2020 and March 2021 in Germany

Gilan et al.
Supplementary Materials

Table S1. Items used to measure study variables.

Mental distress	In the following you will find a description of how you possibly have felt or behaved recently. Please choose the answer that most accurately describes your well-being during the last week.<br>• I felt depressed.<br>• I felt lonely.<br>• I felt hopeful about the future<br>• I felt nervous, anxious, or tense<br>• Thoughts about the pandemic lead to physical reactions such as sweating, breathlessness, dizziness, or increased pulse.
SARS-CoV-2-specific reappraisal	During the coronavirus-pandemic …<br>• I find the necessary ways to keep going.<br>• I know that I will not let it get me down.<br>• I learn important and useful lessons for my life.<br>• I learn possibilities to better cope with it next time.
Social interaction	To what extend do the following statements apply to you in the current situation of limited contact possibilities?<br>• I use phone and digital services to communicate with my family and friends.<br>• I receive support offers from neighbors or relatives.<br>• I offer help to others, for example helping neighbors do their grocery shopping.
Maintaining a daily structure	To what extent do the following statements apply to you in the current situation of limited contact possibilities?<br>• I have a plan for everyday life regarding sleep, work, and physical activities.<br>• I am bored.
Frequency of information intake	• How often do you seek information regarding the coronavirus/SARS-CoV-2?

Note. All reported values are calculated as the mean values of all corresponding items.

Protective and Risk Factors for Mental Distress and Its Impact on Health-Protective Behaviors during the SARS-CoV-2 Pandemic between March 2020 and March 2021 in Germany

Gilan et al.
Supplementary Materials

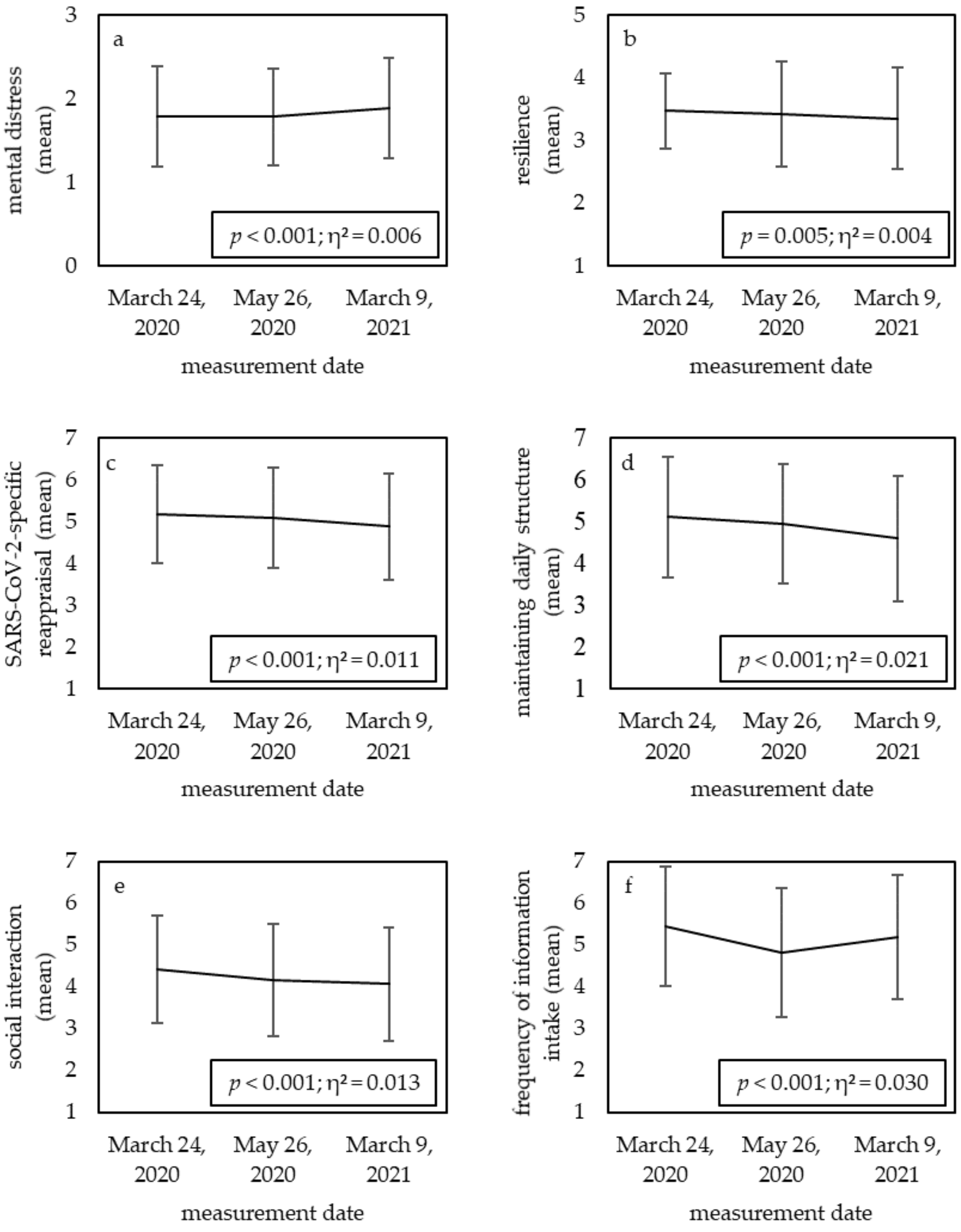

Figure S1. Changes in mental distress (a), resilience (b), SARS-CoV-2-specific reappraisal (c), maintaining daily structure (d), social interaction (e), and frequency of information intake (f) over time. Note: Due to the heterogeneity of variances between samples, differences in SARS-COV-2-specific reappraisal were calculated via the Welch F-test. We applied an α level of 0.005. Effect sizes η^2: small > 0.01, medium > 0.06, large > 0.14.
